# Hereditary transthyretin-related amyloidosis is frequent in polyneuropathy and cardiomyopathy of no obvious aetiology

**DOI:** 10.1080/07853890.2021.1988696

**Published:** 2021-10-16

**Authors:** Volha Skrahina, Ulrike Grittner, Christian Beetz, Thomas Skripuletz, Martin Juenemann, Heidrun H. Krämer, Katrin Hahn, Andreas Rieth, Volker Schaechinger, Monica Patten, Christian Tanislav, Stephan Achenbach, Birgit Assmus, Fabian Knebel, Stefan Gingele, Aliaksandr Skrahin, Jörg Hartkamp, Toni M. Förster, Sabine Roesner, Catarina Pereira, Arndt Rolfs

**Affiliations:** a CENTOGENE GmbH, Rostock, Germany; b Institute of Biometry and Clinical Epidemiology, Charité–Universitätsmedizin Berlin, Berlin, Germany; c Berlin Institute of Health, Berlin, Germany; d Department of Neurology, Hannover Medical School, Hannover, Germany; e Department of Neurology, University Hospital Giessen and Marburg, Giessen, Germany; f Department of Neurology, Charité–Universitätsmedizin Berlin, Berlin, Germany; g Department of Cardiology, Kerckhoff Heart and Lung Center, Bad Nauheim, Germany; h Department of Cardiology, Klinikum Fulda, Fulda, Germany; i Department of Cardiology, University Heart and Vascular Center, Hamburg, Germany; j Department of Neurology, Evangelisches Jung Stilling Krankenhaus GmbH, Siegen, Germany; k Department of Cardiology, Erlangen University Hospital, Erlangen, Germany; l Division of Cardiology and Angiology, University Hospital Giessen and Marburg, Giessen, Germany; m Medizinische Klinik mit Schwerpunkt Kardiologie und Angiologie, Charité–Universitätsmedizin Berlin, Berlin, Germany; n German Centre for Cardiovascular Research, Berlin, Germany; o University Medicine, University Rostock, Rostock, Germany

**Keywords:** Hereditary transthyretin-related amyloidosis, polyneuropathy, cardiomyopathy, genetic testing

## Abstract

**Background:**

Hereditary Transthyretin-Related Amyloidosis, a clinically heterogeneous autosomal dominant disease caused by pathogenic variants in the *TTR* gene, is characterized by the deposition of insoluble misfolded protein fibrils. The diagnosis, especially in non-endemic areas, is typically delayed by 4–5 years; a misdiagnosis due to clinical heterogeneity is common. The study objective was to define the prevalence of Hereditary Transthyretin-Related Amyloidosis in patients with polyneuropathy and/or cardiomyopathy of no obvious aetiology.

**Method:**

A multicenter observational “Epidemiological analysis for the hereditary Transthyretin-Related AMyloidosis”—TRAM study was performed in Germany, Austria, and Switzerland.

**Results:**

A total of 5141 participants were recruited by 50 neurologic and 27 cardiologic specialized centres. Genetic analysis demonstrated a 1.1% Hereditary Transthyretin-Related Amyloidosis positivity rate among patients with polyneuropathy and/or cardiomyopathy of not obvious aetiology. Twenty-one various *TTR* variants (*TTR*-positive) were identified. Body Mass Index was lower in the *TTR*-positive patients as an indicator for the involvement of the autonomic nervous system; the age of onset of clinical manifestations was higher in *TTR*-positive patients. There were no other genotype-phenotype correlations or the prevalence of specific clinical manifestations in *TTR*-positive patients.

**Conclusions:**

Our data support the fact that Hereditary Transthyretin-Related Amyloidosis is underdiagnosed in polyneuropathy and cardiomyopathy patients. Routine implementation of genetic testing is recommended in patients with unexplained polyneuropathy and/or cardiomyopathy to accelerate the earlier diagnosis and the time-sensitive treatment initiation.

**KEY MESSAGES**
- More than 5.000 participants with CM and/or PNP of no obvious aetiology were recruited in the observational “Epidemiological analysis for the hereditary Transthyretin-Related AMyloidosis” TRAM study and screened for pathogenic *TTR* variants.
- The study demonstrated >1% of patients with CM and/or PNP of unclear aetiology are positive for a pathogenic *TTR* variant.
- Routine genetic testing is recommended in patients with unexplained CM and/or PNP to accelerate the initial diagnosis and timely treatment initiation.

## Introduction

Hereditary transthyretin-related amyloidosis (hATTR) is an autosomal dominant condition caused by a pathogenic variant in the *TTR* gene, that is located on chromosome 18q12.1 and comprises 4 exons and 5 introns [1]. Pathogenic variants in the *TTR* gene, coding for the protein transthyretin, result in destabilization and misfolding of this protein. To date, more than 150 *TTR* pathogenic variants are described which cause amyloid formation [2]. Still, the penetrance is not clear for most of the variants. hATTR has been considered an endemic disease with early or late-onset in Japan, Sweden, and Portugal.

hATTR manifests with a variety of clinical presentations of which cardiomyopathy (CM) and polyneuropathy (PNP) are the most common [3,4]. Due to the heterogeneity of the phenotype, the diagnosis is delayed by 4–5 years, especially in non-endemic areas, and the misdiagnosis is a frequent burden for such patients [5–7].

hATTR should be considered in older persons who have been hospitalized for heart failure, elevated troponin or N-terminal pro-brain natriuretic peptide (NT-proBNP) and intolerance of angiotensin-converting enzyme (ACE) inhibitors, angiotensin receptor blockers, or β blockers [8]. Lumbar spinal stenosis [9], previous orthopaedic procedures [10], spontaneous biceps tendon rupture [11], carpal tunnel syndrome [12] may be early indicators of hATTR in patients with progressive disabilities. The most typical presentation of hATTR PNP is a progressive, length-dependent, mixed sensory and motor peripheral polyneuropathy, which usually begins with the loss of thermal and pain sensation in the feet, slowly ascends the limbs, and is typically associated with variable autonomic disturbances [1].

Usually, hATTR diagnosis requires the histologic demonstration of amyloid deposits in biopsy specimens. However, initial genetic testing is a fast, cheap (due to the small gene size), and reliable analysis to prove for hATTR. The most prevalent variant associated with amyloid PNP is NM_000371.3:c.148G > A (p.Val50Met) [13,14]. Affected patients with this genotype display a variable disease penetrance which is influenced by origin, gender, parental gene transmission, and age [1,15]. The most common variant involved in the cardiac form of hATTR is NM_000371.3:c.424G > A (p.Val142Ile) [16,17]. The NM_000371.3:c.238A > G (p.Thr80Ala) variant is another frequent cause of amyloid PNP and CM [18].

Within Europe, the incidence of hATTR is highly variable. There are large areas in Portugal and Sweden where the disease is endemic. The described prevalence for Portugal is 192 per 1,000,000 and for Sweden it is 26 per 1,000,000 inhabitants [13]. Smaller endemic areas have been identified in Cyprus (56 per 1,000,000 inhabitants) and Majorca (50 per 1,000,000 inhabitants) [19,20]. In the rest of Europe, cases of hATTR are mainly seen as sporadic [21]. However, up to now, only a few systematic prospective studies describing the hATTR prevalence among larger groups of patients have been published [22,23].

The aim of the study was therefore based on the NGS-sequencing of the *TTR* gene to investigate the hATTR prevalence in a large risk group: patients with PNP and/or CM of no obvious aetiology. In the case of the detection of a pathogenic variant in *TTR*, patients obtain the diagnosis for hATTR for which causative treatments are available.

## Methods

The “Epidemiological analysis for the hereditary TransthyRetin-related AMyloidosis” (TRAM) study was approved by Ethics Committee at the Medical Faculty of the University of Rostock: approval A 2018-0069, 19.04.2018. Ethics approval was obtained for each participating centre in Germany, Austria, and Switzerland. A list of all Ethics committees that have approved the study in all three countries is provided (Supplementary Information).

The study was registered in ClinicalTrials.gov with the number NCT03237494.

### Study design

The TRAM study is an observational epidemiological study that has been conducted in Germany, Austria, and Switzerland. Participants with PNP and/or CM of no obvious aetiology from 77 centres with either a neurologic (*n* = 50) or cardiologic (*n* = 27) specialty were enrolled (Supplementary Table 1S).

### Eligibility criteria

The inclusion criteria are as follows: (1) informed consent is obtained from the participant; (2) the participant is diagnosed with CM and/or PNP of no obvious aetiology; (3) the participant is aged between 18 and 85 years; (4) the participant has not undergone chemotherapy for any solid or hematological malignancies. There are no specific exclusion criteria.

All enrolled participants have visited the study site one time. At this visit, a Case Report Form (CRF) was completed, and a blood sample was drawn and applied on a Dried Blood Spot (DBS) filter CentoCard^®^. The blood sample was used for genetic analysis to screen for hATTR.

### Clinical information

All clinical information is documented by the physician in an electronic CRF that asks for physical examinations, demographic details, current symptoms, medication, and family history. The following current symptoms and concomitant medication were questioned by the physician with symptoms of PNP grouped at the top and symptoms of CM grouped at the bottom of the table (Table 1). Height and weight were used to calculate the Body Mass Index (BMI) of each subject and echocardiography was used to assess left ventricular thickness. All CRFs are stored with appropriate measures to maintain data privacy regulations (all data are encrypted in transit with TLS1.2). CENTOGENE GmbH operates core services in an external Data Centre. Based on multiple certifications, the Data Centre provider ensures compliance to applicable international legislation (e.g. GDPR and HIPPA) and a high level of IT Security and Business Continuity. The Data Centre is certified for different quality standards: ISO 9001—Quality Management System; ISO 27001—Information Security Management System.

**Table 1.** Current symptoms for polyneuropathy and cardiomyopathy of undetermined aetiology.

Does the patient have any of the following?	Number of patients with information	Present *n* (%)
Reduced ability to sense temperature	4016	1090 (27.1)
Numbness	4017	2177 (54.2)
Tingling	4016	2041 (50.8)
“Burning feet”	4017	1410 (35.1)
Pain	4016	1912 (47.6)
Muscle atrophy in the leg	4017	775 (19.3)
History of carpal tunnel syndrome	4016	704 (17.5)
Allodynia	4017	527 (13.1)
Muscle weakness in the leg	4017	1470 (36.6)
Difficulty urinating or holding the urine	4016	638 (15.9)
Dizziness or fainting upon standing	4015	1468 (36.6)
Inability to obtain or maintain an erection	2514 males	515 (20.5)
Bouts of constipation that alternate with diarrhoea	4017	312 (7.8)
Unintentional weight loss	4017	435 (10.8)
Anaemia	4017	287 (7.1)
Diarrhoea	4017	481 (12.0)
Constipation	4016	583 (14.5)
Shortness of breath	4017	1551 (38.6)
Water accumulation in the ancles and lower limbs	4017	1209 (30.1)
Dyshidrosis	4017	662 (16.5)
Chest pain	4015	652 (16.2)
Palpitation	4016	1122 (27.9)
Arrhythmia	4015	1014 (25.3)
Enlargement of heart cavities	4015	830 (20.7)
Enlargement of heart muscle	4017	1025 (25.5)
Abnormal (fluttering) heartbeat	4015	1011 (25.2)

### Genetic testing

All enrolled participants have undergone genetic testing for hATTR. DNA from the CentoCard^®^ was extracted using the Extracta DNA Prep Kit according to the manufacturer’s instructions (QuantaBio). Afterward, the concentration of the DNA was measured with the Quantifluor system in a Fluoroskan Ascent™ FL Microplate Fluorometer (ThermoFisher). The extracted DNA was used in a multiplex PCR generating 12 *TTR*-specific amplicons (Primer sequences and PCR program in Annexe 1). PCR products from both pools are pooled together and purified using AMPure XP magnetic beads (Beckman Coulter) in a 1:1 PCR product: beads ratio to remove unbound primers. A second PCR amplification is performed adding Illumina-compatible and patient-specific barcoded index primers to each amplicon. After another purification round, the samples are pooled together and sequenced *via* Next-Generation Sequencing with a 300 cycle v2 MiSeq kit and a MiSeq Instrument (Illumina Inc.). The resulting 150 bp long paired-end reads are aligned with bioinformatic methods and compared to the reference sequence *TTR*: NM_000371.3 to identify single nucleotide variants (SNVs). All variants are classified according to the American College of Medical Genetics (ACMG) into Class 1 (pathogenic), Class 2 (likely pathogenic), Class 3 (variance of uncertain significance, VUS), Class 4 (likely benign), and Class 5 (benign), only class 1, 2, and 3 are reported *via* a medical report. These classifications were assessed using existing information from HGMD^®^ [24], ClinVar [25], and the in-house platform CentoMD [26].

In cases where no relevant SNVs were detected by the above described NGS, an in-house developed deletion/duplication analysis was conducted, using endpoint quantification by Real-Time PCR. These RT-PCRs are conducted on a LightCycler 480 II (Roche) with VeriQuest Fast SYBR Green qPCR Master Mix (ThermoFisher) and specifically designed primers targeting each *TTR* exon. The expression of each *TTR* exon was evaluated by a relative quantification analysis (ΔΔCT-Method) with *GUSB* serving as reference gene, and thereby showing whether a deletion or duplication is present.

### Statistical methods

Descriptive statistical analysis methods (absolute and relative frequencies, mean, standard deviation, median, and range) were used to describe the total study population and to compare *TTR*-positive and *TTR*-negative patients. As exploratory statistical tests, *χ*^2^-Test was used to test differences in nominal data, and *t*-test for independent samples was used to test continuous variables between *TTR*-positive and *TTR*-negative patients. We also report effect sizes and 95% confidence intervals (CI), namely Cohen’s *d* for continuous data and Cramer’s *V* for nominal data. As statistical Software SPSS (IBM Corp. Released 2019) [27] and R [28], package effect size [29] was used. A two-sided significance level of *α* = 0.05 was considered. However, since no adjustment for multiple testing was applied in this exploratory study, interpretation of *p*-values should be done cautiously. Interpretation of results is therefore primarily based on effect sizes and 95% CI.

## Results

### Study participants

The participants (*n* = 5213) with PNP and/or CM of not obvious aetiology were invited to participate in the study, 72 were further excluded due to incomplete/insufficient clinical information or the consent withdrawal. Of those included (*n* = 5141), 4925 were enrolled in Germany, 111 in Switzerland, and 105 in Austria. Males and females accounted for 61.9 and 38.1% of the participants, respectively. The mean age at enrolment was 60.2 years (SD: 14.3; median 61; range 18–85), while the mean age at disease onset was 55.6 years (SD: 15.7; median 57; range 1–85). A total 2850 patients (55.4%) suffered from CM and, 1221 patients (23.8%) from PNP while 1070 patients (20.8%) suffered from both, CM and PNP.

### Genetic findings

**Figure 1.**
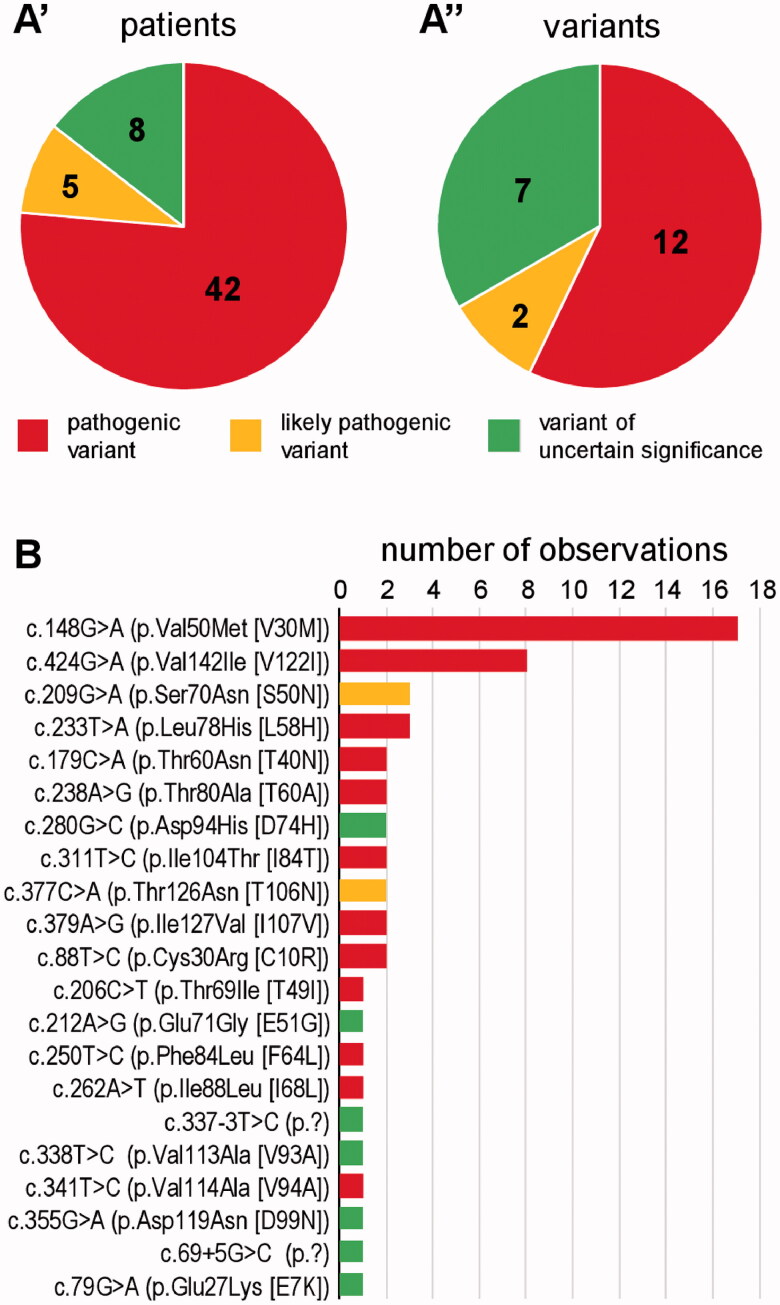
*TTR* variants as identified by the present study. (A) Fraction of the three relevant classes amongst patients (A’) and variants (A”). Absolute numbers are provided within the charts. (B) Number of observations of individual variants. Colour shade-coding as in (A).

Sequencing of the *TTR* gene in the 5141 study participants revealed the presence of a reportable heterozygous variant in 55 subjects (1.1% of all; subsequently referred to as *TTR-*positives). Forty-two of these (76.4%) were diagnosed with Class 1, five (9.1%) with Class 2, and eight (14.5%) with Class 3 variant (Figure 1(A’)). There was a total of 21 distinct variants; twelve pathogenic, two likely pathogenic, and seven of uncertain significance (Figure 1(A”)). Nineteen variants were predicted to result in missense alteration at protein level; two affect intronic splice motifs. The two variants, c.148G > A (p.Val50Met) and c.424G > A (p.Val142Ile), were outstandingly frequent, while all other variants were observed between one and three times each (Figure 1(B)).

The clinical and demographic characteristics of each *TTR* variant previously described in the literature [2,25,30–40] and found in our study are shown in Table 2.

**Table 2.** *TTR* heterozygous variants in patients with PNP and/or CM.

	Literature		TRAM study				
Variant name	Phenotype	Ethnicity	*n*	m/f	Age of onset, years	Phenotype	Ethnicity
NM_000371.3:							
c.148G > A (p.Val50Met)	AN E LM PNP	AM CN JP EUR DE ES FR IT PT SE AT	17	11/6	15–78	CM-2 PNP-10	DE
						CM/PNP-5	
c.424G > A (p.Val142Ile)	H	AFR AM FR PT IT AT	8	8/0	30**–**78	CM-5 PNP-1	DE AT
						CM/PNP-2	
c.233T > A (p.Leu78His)	H CTS	AM DE	3	3/0	57, 62, 63	PNP-3	DE
c.179C > A (p.Thr60Asn)	H AN PNP	DE	2	1/1	71, 75	CM/PNP-2	DE
c.238A > G (p.Thr80Ala)	CTS H PNP	AT DE IE DON BR AM AP SE	2	1/1	68, 72	CM-1 PNP-1	DE
c.311T > C (p.Ile104Thr)	H PNP	DE BR	2	1/1	59, 68	CM/PNP-2	DE
c.377C > A (p.Thr126Asn)	–	–	2	1/1	66, 73	CM-1 CM/PNP-1	DE
c.379A > G (p.Ile127Val)	CTS H PNP	DE AM	2	2/0	55, 70	PNP-2	DE
c.88T > C (p.Cys30Arg)	AN E H PNP	AM HU	2	1/1	76, 78	PNP-1 CM-1	DE
c.206C > T (p.Thr69Ile)	H PNP	JP	1	1/0	58	CM/PNP	AT
c.212A > G (p.Glu71Gly)	H	AM	1	1/0	42	PNP	DE
c.250T > C (p.Phe84Leu)	CTS H PNP	IT AM	1	1/0	66	PNP	DE
c.262A > T/C (p.Ile88Leu)	H	DE AM IT	1	1/0	82	CM/PNP	DE
c.341T > C (p.Val114Ala)	AN H PNP	DE GR CYP AT	1	0/1	71	CM/PNP	DE
c.337-3T > C	H	–	1	0/1	58	CM	DE
c.209G > A (p.Ser70Asn)	NA	–	3	1/2	63, 65, 67	PNP-2 CM/PNP-1	DE
c.280G > C (p.Asp94His)	NA	DE	2	2/0	58, 78	PNP-2	DE
c.338T > C (p.Val113Ala)	NA	–	1	0/1	34	PNP	DE
c.355G > A (p.Asp119Asn)	NA	–	1	1/0	68	PNP	DE
c.79G > A (p.Glu27Lys)	NA	–	1	1/0	63	PNP	DE
c.69 + 5G > C	NA	–	1	1/0	43	PNP	DE

AN: autonomic neuropathy; CTS: carpal tunnel syndrome; E: eye; H: heart; LM: leptomeningeal; NA: non-amyloidogenic; CM: cardiomyopathy; PNP: polyneuropathy; AFR: African; AM: American; AP: Appalachian; AT: Austrian; BR: British; CN: Chinese; CYP: Cypriot; DE: German; DON: Donegan; EUR: European; ES: Spanish; FR: French; GR: Greek; IT: Italian; IE: Irish; PT: Portuguese; SE: Swedish; HU: Hungarian.

### Characteristics of TTR-positive vs. TTR-negative

**Figure 2.**
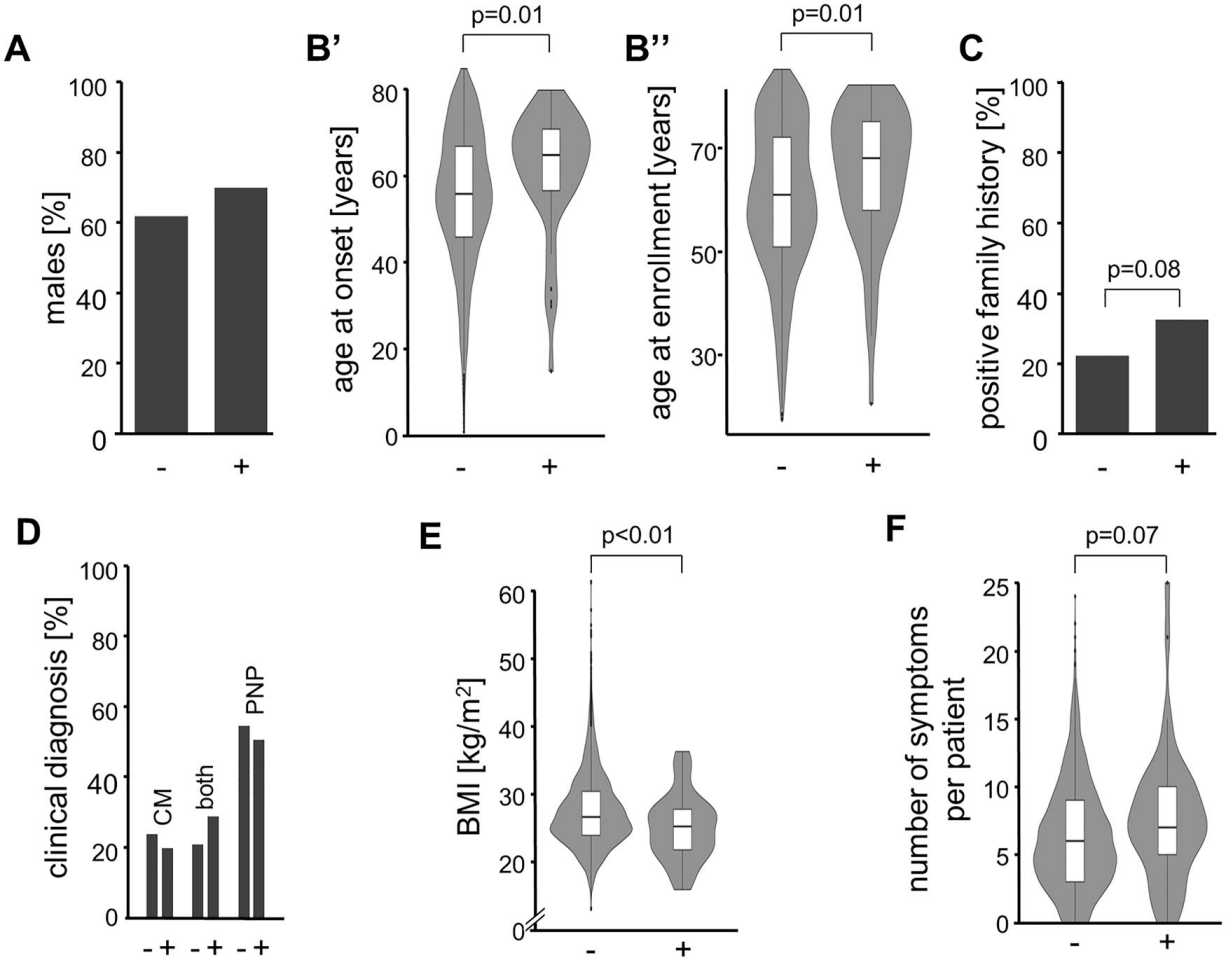
Comparison of *TTR*-positive and *TTR*-negative study participants (*TTR*-status on the *x*-axis in all charts). (A) Gender. (B) Ages at disease onset and enrolment. (C) Family history. (D) Manifestation as cardiomyopathy (CM), polyneuropathy (PNP), or both. (E) Body Mass Index (BMI). (F) Number of symptoms.

**Figure 3.**
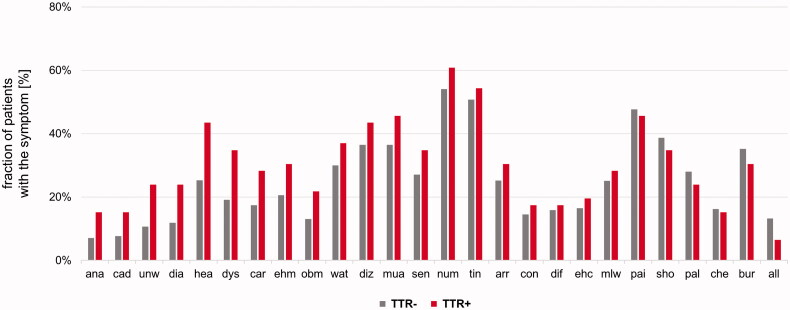
Frequency of individual symptoms with TTR-negative (TTR−) depicted in grey and TTR-positive (TTR+) in red colour. Symptoms that show discrepancies between *TTR*-negative and *TTR*-positive study participants are depicted on the left. Symptoms in alphabetical order: All: Allodynia; ana: Anaemia; arr: Arrhythmia; bur: “burning feet”; car: History of carpal tunnel syndrome; che: Chest pain; cad: Bouts of constipation that alternate with diarrhoea; con: Constipation; dia: Diarrhoea; dif: Difficulty urinating or holding urine; diz: Dizziness or fainting upon standing; dys: Dyshidrosis; ehc: Enlargement of heart cavities; ehm: Enlargement of heart muscle; hea: Abnormal (fluttering) heartbeat; mlw: Muscle weakness in the legs; mua: Muscle atrophy in the legs; num: Numbness; obm: Inability to obtain or maintain an erection; pai: Pain; pal: Palpitation; sen: Reduced ability to sense temperature; sho: Shortness of breath; tin: Tingling; unw: Unintentional weight loss; wat: Water accumulation in the ankles and lower limbs.

In the first step, the study participants were genetically stratified into 55 *TTR*-positive *vs.* 5086 *TTR*-negative, next test was performed whether these subgroups differed in any of the available clinical and demographic parameters. The distribution of sex was similar in *TTR*-positive and *TTR*-negative with a slightly higher proportion of males in the *TTR*-positive [69 *vs.* 62% males, *p* = .269, Cramer’s *V*: 0.02, 95%CI: 0–0.04, Figure 2(A)]. Both, age at onset and age at enrolment, however, were higher in *TTR*-positive [mean difference for the age of onset: 6 years, 95%CI: 1–10 years, Cohen’s *d*: 0.39, 95%CI: 0.09–0.69, *p* = .006; mean difference for the age of enrolment: 5 years, 95%CI: 1–8 years, Cohen’s *d*: 0.34, 95%CI: 0.08–0.61, *p* = .008, Figures 2(B’,B”)]. Four *TTR*-positive patients had disease onset before 40 years: 15 and 31 years with c.148G > A (p.Val50Met), 30 years with c.424G > A (p.Val142Ile), and 34 years with c.338T > C (p.Val113Ala). *TTR*-positive patients reported more often a family history of CM or PNP than *TTR*-negative individuals (33 *vs.* 22%, *p* = .085, Cramer’s *V*: 0.03, 95%CI: 0–0.6) (Figure 2(C)). While a clinical picture consistent with a diagnosis of both CM and PNP was more often seen in *TTR*-positive than in *TTR*-negative (29 *vs.* 21%), this difference was not substantial [*p* = .306, Cramer’s *V*: 0.02, 95%CI: 0–0.05, Figure 2(D)]. We did, however, find that the BMI was lower in *TTR-*positive [mean difference: 2.4 kg/m^2^, 95% CI: 1.0–3.9 kg/m^2^, *p* = .002, Cohen’s *d*: 0.44, 95% CI: 0.15–0.74, Figure 2(E)]. In contrast, none of the 26 individually documented symptoms was substantially more frequent in either of the two groups (Figure 3). However, as odds ratios (*TTR*-positive over *TTR*-negative) were still >1 for 20 of the 26 symptoms [e.g. anaemia, history of carpal tunnel syndrome, diarrhoea, dyshidrosis, abnormal (fluttering) heartbeat, and unintentional weight loss], we also compared the average number of symptoms *per* subject between the groups. Although not substantially but the symptoms load was higher in the *TTR*-positive group [mean difference: 1.3, 95% CI: −0.1–2.7, *p* = .074, Cohen’s *d*: 0.34, 95%CI: 0.05–0.63, Figure 2(F)]. Decision-trees analyses that considered the absence/presence of the individual symptoms did not identify a model in which both sensitivity and positive prediction value were >50% (data not shown).

## Discussion

The *TTR* variants c.148G > A (p.Val50Met)—31%, c.424G > A (p.Val142Ile)—15%, and c.209G > A (p.Ser70Asn)—5%, c.233T > A (p.Leu78His)—5% were the most frequent identified in our study. Among the participants registered in Amyloidosis Outcomes Survey (THAOS) registry c.148G > A (p.Val50Met) variant was the most frequent (73.6%), particularly in Portugal and Sweden, followed by c.262A > T (p.Ile88Leu)—2.2%, with most subjects from Italy [31]. The *TTR* variant c.424G > A (p.Val142Ile) was only in fourth place in prevalence in Europe (1.4%) (with most patients from France), however the most frequent in the USA among African Americans [41]. The c.238A > G (p.Thr80Ala) variant, most frequent in Northwestern Ireland [34], in our study, occurred in 2 (4%) subjects. European *TTR* variants, c.325G > C (p.Glu109Gln)—1.9%, c.290C > A (p.Ser97Tyr)—1.3%, c.200G > C (p.Gly67Ala)—1.2%, c.118G > A (p.Val40Ile)—0.9%, c.391C > A (p.Leu131Met)—0.9% [31] were not found in our study. The variants c.424G > A (p.Val142Ile), c.88T > C (p.Cys30Arg), c.206C > T (p.Thr69Ile), c.212A > G (p.Glu71Gly), and c.250T > C (p.Phe84Leu) identified in our study were not previously described in Germany. Gender distribution in our study is in line with the majority of previous studies where proportions of men are reported from 61 to 86% [42]. However, only 52% of the male are among hATTR patients in the THAOS registry [31].

The differences between our data and the data of the THAOS registry, including its German cohort, can be explained by different mosaic geographic (within Germany) distributions of hATTR participants both by prevalence and by variants.

Clinical phenotype of hATTR in the THAOS subjects was CM in 14.9%, CM + PNP in 21.1%, and PNP in 64.0%. The proportion of the PNP phenotype in the cohort was driven by Portugal, Sweden, 79.6 and 64.1%, respectively. In Germany and Denmark most subjects had CM, 51 and 78%, respectively [31], which is in contrast to our study revealing the PNP phenotype in 51% of participants, CM phenotype in 20%, and mixed presentation in 29% (Figure 2(D)). This difference can be explained as the THAOS study included a large portion of wild-type ATTR which is predominantly associated with CM, whereas wild-type ATTR subjects were excluded from our study.

In our study c.148G > A (p.Val50Met) historically known for its PNP clinical pattern [2,30–32] caused pure CM in 2 (12%) cases and CM + PNP clinical presentation in 5 (29%) cases. Known as CM variant, c.424G > A (p.Val142Ile) [2,31,32,34,41], presented in our study as PNP in 1 (13%) case, mixed CM/PNP pattern in 2 (25%) cases. All three participants with the variant c.233T > A (p.Leu78His) presented with PNP, instead of CM as described in the literature [2,32]. In the majority of c.148G > A (p.Val50Met) subjects in Portugal the onset is typically at 30–40 years, “early” onset, while the majority of patients with c.148G > A (p.Val50Met) in Sweden has the onset after 50 years “late” onset [21]. A “late” and “early” distribution of the onset among patients with the c.148G > A (p.Val50Met) variant was reported in Japan [43]. Of 24 subjects from Germany with c.148G > A (p.Val50Met) variant in THAOS registry 13 (54%) had “early” onset, 11 (46%) “late” onset [31]. Two out of 17 (17%) of our participants with c.148G > A (p.Val50Met) variant had “early” Portuguese-type, the other (83%) had “late” Swedish-type of disease onset. Although it has been reported previously that the early onset is not typical for non-c.148G > A (p.Val50Met) variants [44,45], in our study two subjects with c.424G > A (p.Val142Ile) and c.338T > C (p.Val113Ala) variants had the onset at 30 and 34 years, accordingly. Such is highly likely due to various screening strategies: Testing the general population *vs.* risk cohort (CM and/or PNP individuals). Thus, the TRAM study complements the previous studies in terms of clinical manifestations and age of onset of various variants of hATTR.

A substantially lower BMI in the *TTR*-positive group was detected. Autonomic manifestations are present in 50–80% of hATTR patients, most frequently patients demonstrate diarrhoea, constipation, postural hypotension, urinary incontinence, or erectile dysfunction [46]. Different variants might reflect different likelihoods for autonomic dysfunction. In the THAOS registry, the prevalence of autonomic dysfunction was higher in patients with c.148G > A (p.Val50Met) [31]. Modified BMI (mBMI, BMI multiplied by serum albumin level to compensate for edema) value has demonstrated a strong correlation with survival in patients with c.148G > A (p.Val50Met) variant [47]. The difference in BMI between *TTR* positive and negative patients is because the spectrum of “unknown” diseases manifested by PNP and CM (*TTR* negative) is not characterized by the involvement of the autonomic nervous system.

### Strengths and limitations

Our study is the largest systematic cohort in an hATTR risk group with more than 5000 participants demonstrating CM, PNP, and both CM/PNP of no obvious aetiology.

A potential limitation of our study is the lack of a longitudinal component to investigate how clinical symptoms progress over the time or with the treatment.

### Conclusions and health care implications

Our study showed >1% of patients with CM and/or PNP of unclear aetiology are positive for a pathogenic variant in *TTR* gene. This confirms the need for mandatory genetic testing and counselling for all patients in this group.

## Supplementary Material

Supplemental Material

## Data Availability

The data that support the findings of this study are available upon reasonable request from the corresponding author, J.H. (email: joerg.hartkamp@centogene.com). The data are not publicly available due to containing information that could compromise the privacy of research participants.

## References

[1] Planté-Bordeneuve V, Said G. Familial amyloid polyneuropathy. Lancet Neurol. 2011;10(12):1086–1097. 22094129 10.1016/S1474-4422(11)70246-0

[2] Rowczenio DM, Noor I, Gillmore JD, et al. Online registry for mutations in hereditary amyloidosis including nomenclature recommendations. Hum Mutat. 2014;35(9):E2403–E2412. 25044787 10.1002/humu.22619

[3] Adams D, Ando Y, Beirão JM, et al. Expert consensus recommendations to improve diagnosis of ATTR amyloidosis with polyneuropathy. J Neurol. 2021;268(6):2109–2122. 31907599 10.1007/s00415-019-09688-0 PMC8179912

[4] Adams D, Koike H, Slama M, et al. Hereditary transthyretin amyloidosis: a model of medical progress for a fatal disease. Nat Rev Neurol. 2019;15(7):387–404. 31209302 10.1038/s41582-019-0210-4

[5] Lozeron P, Lacroix C, Theaudin M, et al. An amyotrophic lateral sclerosis-like syndrome revealing an amyloid polyneuropathy associated with a novel transthyretin mutation. Amyloid. 2013;20(3):188–192. 23914756 10.3109/13506129.2013.818535

[6] Lozeron P, Mariani LL, Dodet P, et al. Transthyretin amyloid polyneuropathies mimicking a demyelinating polyneuropathy. Neurology. 2018;91(2):e143–e152. 29907605 10.1212/WNL.0000000000005777

[7] Cortese A, Vegezzi E, Lozza A, et al. Diagnostic challenges in hereditary transthyretin amyloidosis with polyneuropathy: avoiding misdiagnosis of a treatable hereditary neuropathy. J Neurol Neurosurg Psychiatry. 2017;88(5):457–458. 28188196 10.1136/jnnp-2016-315262 PMC5529976

[8] Maurer MS, Bokhari S, Damy T, et al. Expert consensus recommendations for the suspicion and diagnosis of transthyretin cardiac amyloidosis. Circ Heart Fail. 2019;12(9):e006075. 31480867 10.1161/CIRCHEARTFAILURE.119.006075 PMC6736650

[9] Eldhagen P, Berg S, Lund LH, et al. Transthyretin amyloid deposits in lumbar spinal stenosis and assessment of signs of systemic amyloidosis. J Intern Med. 2021;289(6):895–905. 33274477 10.1111/joim.13222 PMC8248398

[10] Rubin J, Alvarez J, Teruya S, et al. Hip and knee arthroplasty are common among patients with transthyretin cardiac amyloidosis, occurring years before cardiac amyloid diagnosis: can we identify affected patients earlier? Amyloid. 2017;24(4):226–230. 28906148 10.1080/13506129.2017.1375908

[11] Geller HI, Singh A, Alexander KM, et al. Association between ruptured distal biceps tendon and wild-type transthyretin cardiac amyloidosis. JAMA. 2017;318(10):962–963. 28898370 10.1001/jama.2017.9236 PMC5818850

[12] Nakagawa M, Sekijima Y, Yazaki M, et al. Carpal tunnel syndrome: a common initial symptom of systemic wild-type ATTR (ATTRwt) amyloidosis. Amyloid. 2016;23(1):58–63. 26852880 10.3109/13506129.2015.1135792

[13] Ando Y, Nakamura M, Araki S. Transthyretin-related familial amyloidotic polyneuropathy. Arch Neurol. 2005;62(7):1057–1062. 16009758 10.1001/archneur.62.7.1057

[14] Olsson M, Jonasson J, Cederquist K, et al. Frequency of the transthyretin Val30Met mutation in the Northern Swedish population. Amyloid. 2014;21(1):18–20. 24555660 10.3109/13506129.2013.860027

[15] Hellman U, Alarcon F, Lundgren HE, et al. Heterogeneity of penetrance in familial amyloid polyneuropathy, ATTR Val30Met, in the Swedish population. Amyloid. 2008;15(3):181–186. 18925456 10.1080/13506120802193720 PMC2738945

[16] Damrauer SM, Chaudhary K, Cho JH, et al. Association of the V122I hereditary transthyretin amyloidosis genetic variant with heart failure among individuals of African or Hispanic/Latino ancestry. JAMA. 2019;322(22):2191–2202. 31821430 10.1001/jama.2019.17935 PMC7081752

[17] Alexander KM, Falk RH. V122I TTR cardiac amyloidosis in patients of African descent: recognizing a missed disease or the dog that didn't bark? Circ Heart Fail. 2016;9(9):e003489. 27618856 10.1161/CIRCHEARTFAILURE.116.003489

[18] Miller SR, Sekijima Y, Kelly JW. Native state stabilization by NSAIDs inhibits transthyretin amyloidogenesis from the most common familial disease variants. Lab Invest. 2004;84(5):545–552. 14968122 10.1038/labinvest.3700059

[19] Reinés JB, Vera TR, Martín MU, et al. Epidemiology of transthyretin-associated familial amyloid polyneuropathy in the Majorcan area: Son Llàtzer Hospital descriptive study. Orphanet J Rare Dis. 2014;9:29. 24572009 10.1186/1750-1172-9-29 PMC3941569

[20] Dardiotis E, Koutsou P, Papanicolaou EZ, et al. Epidemiological, clinical and genetic study of familial amyloidotic polyneuropathy in Cyprus. Amyloid. 2009;16(1):32–37. 19291512 10.1080/13506120802676948

[21] Parman Y, Adams D, Obici L, et al. Sixty years of transthyretin familial amyloid polyneuropathy (TTR-FAP) in Europe: where are we now? A European network approach to defining the epidemiology and management patterns for TTR-FAP. Curr Opin Neurol. 2016;29(Suppl 1):S3–S13. 26734951 10.1097/WCO.0000000000000288 PMC4739317

[22] Lopes LR, Futema M, Akhtar MM, et al. Prevalence of TTR variants detected by whole-exome sequencing in hypertrophic cardiomyopathy. Amyloid. 2019;26(4):243–247. 31554435 10.1080/13506129.2019.1665996

[23] Damy T, Costes B, Hagege AA, et al. Prevalence and clinical phenotype of hereditary transthyretin amyloid cardiomyopathy in patients with increased left ventricular wall thickness. Eur Heart J. 2016;37(23):1826–1834. 26537620 10.1093/eurheartj/ehv583

[24] Human gene mutation database.2020.4; 2020 [cited 2020 Dec 18]. Available from: https://digitalinsights.qiagen.com/

[25] ClinVar - NCBI; https://www.ncbi.nlm.nih.gov/clinvar/

[26] Trujillano D, Oprea GE, Schmitz Y, et al. A comprehensive global genotype-phenotype database for rare diseases. Mol Genet Genomic Med. 2017;5(1):66–75. 28116331 10.1002/mgg3.262 PMC5241210

[27] IBM Corp. IBM SPSS statistics for windows, Version 26.0. Armonk (NY): IBM Corp; 2019.

[28] R Core Team. R: a language and environment for statistical computing. Vienna: R Foundation for Statistical Computing; 2017 [cited 2020 Dec 18]. Available from: https://www.R-project.org/

[29] Ben-Shachar Makowski Lüdecke Compute and interpret indices of effect size. CRAN; 2020 [cited 2020 Dec 18]. Available from: https://github.com/easystats/effectsize

[30] Rapezzi C, Quarta CC, Obici L, et al. Disease profile and differential diagnosis of hereditary transthyretin-related amyloidosis with exclusively cardiac phenotype: an Italian perspective. Eur Heart J. 2013;34(7):520–528. 22745357 10.1093/eurheartj/ehs123

[31] Damy T, Kristen AV, Suhr OB, et al. Transthyretin cardiac amyloidosis in continental Western Europe: an insight through the transthyretin amyloidosis outcomes survey (THAOS). Eur Heart J. 2019. 10.1093/eurheartj/ehz173 PMC8825236 30938420

[32] OMIM. Online Mendelian Inheritance in Man; 2020 [cited 2020 Dec 18]. Available from: https://omim.org/allelicVariants/

[33] Hinderhofer K, Obermaier C, Hegenbart U, et al. New sequence variants in patients affected by amyloidosis show transthyretin instability by isoelectric focusing. Amyloid. 2019;26(2):85–93. 10.1080/13506129.2019.1598358 31074293

[34] Reilly MM, Staunton H, Harding AE. Familial amyloid polyneuropathy (TTR ala 60) in North West Ireland: a clinical, genetic, and epidemiological study. J Neurol Neurosurg Psychiatry. 1995;59(1):45–49. 7608709 10.1136/jnnp.59.1.45 PMC1073600

[35] Sattianayagam PT, Hahn AF, Whelan CJ, et al. Cardiac phenotype and clinical outcome of familial amyloid polyneuropathy associated with transthyretin alanine 60 variant. Eur Heart J. 2012;33(9):1120–1127. 21992998 10.1093/eurheartj/ehr383

[36] Staunton H, Davis MB, Guiloff RJ, et al. Irish (Donegal) amyloidosis is associated with the transthyretinALA60 (Appalachian) variant. Brain. 1991;114(6):2675–2679. 1664269 10.1093/brain/114.6.2675

[37] Uemichi T, Murrell JR, Zeldenrust S, et al. A new mutant transthyretin (Arg 10) associated with familial amyloid polyneuropathy. J Med Genet. 1992;29(12):888–891. 1362222 10.1136/jmg.29.12.888 PMC1016207

[38] Nakamura M, Yamashita T, Ando Y, et al. Identification of a new transthyretin variant (Ile49) in familial amyloidotic polyneuropathy using electrospray ionization mass spectrometry and nonisotopic RNase cleavage assay. Hum Hered. 1999;49(4):186–189. 10436378 10.1159/000022872

[39] Almeida MR, Hesse A, Steinmetz A, et al. Transthyretin leu 68 in a form of cardiac amyloidosis. Basic Res Cardiol. 1991;86(6):567–571. 1786038 10.1007/BF02190707

[40] Kristen AV, Ehlermann P, Helmke B, et al. Transthyretin valine-94-alanine, a novel variant associated with late-onset systemic amyloidosis with cardiac involvement. Amyloid. 2007;14(4):283–287. 17968688 10.1080/13506120701616383

[41] Jacobson DR, Alexander AA, Tagoe C, et al. Prevalence of the amyloidogenic transthyretin (TTR) V122I allele in 14 333 African-Americans. Amyloid. 2015;22(3):171–174. 26123279 10.3109/13506129.2015.1051219

[42] Waddington-Cruz M, Schmidt H, Botteman MF, et al. Epidemiological and clinical characteristics of symptomatic hereditary transthyretin amyloid polyneuropathy: a global case series. Orphanet J Rare Dis. 2019;14(1):34. 30736835 10.1186/s13023-019-1000-1 PMC6368811

[43] Sekijima Y, Ueda M, Koike H, et al. Diagnosis and management of transthyretin familial amyloid polyneuropathy in Japan: red-flag symptom clusters and treatment algorithm. Orphanet J Rare Dis. 2018;13(1):6. 29343286 10.1186/s13023-017-0726-x PMC5773042

[44] Mazzeo A, Russo M, Di Bella G, et al. Transthyretin-related familial amyloid polyneuropathy (TTR-FAP): a single-center experience in Sicily, an Italian endemic area. J Neuromuscul Dis. 2015;2(s2):S39–S48. 10.3233/JND-150091 PMC5271421 27858761

[45] Meng LC, Lyu H, Zhang W, et al. Hereditary transthyretin amyloidosis in eight Chinese families. Chin Med J. 2015;128(21):2902–2905. 26521788 10.4103/0366-6999.168048 PMC4756886

[46] Gonzalez-Duarte A, Valdés-Ferrer SI, Cantú-Brito C. Characteristics and natural history of autonomic involvement in hereditary ATTR amyloidosis: a systematic review. Clin Auton Res. 2019;29(Suppl 1):1–9. 10.1007/s10286-019-00630-y PMC6763513 31473866

[47] Suhr O, Danielsson A, Holmgren G, et al. Malnutrition and gastrointestinal dysfunction as prognostic factors for survival in familial amyloidotic polyneuropathy. J Intern Med. 1994;235(5):479–485. 8182405 10.1111/j.1365-2796.1994.tb01106.x

