# Supplementary material for: Hereditary transthyretin-related amyloidosis is frequent in polyneuropathy and cardiomyopathy of no obvious aetiology
Source: Ann Med. 2021 Oct 16;53(1):1787–96. doi: 10.1080/07853890.2021.1988696 (PMC8525987; doi:10.1080/07853890.2021.1988696)
Supplement: Supplemental Material [file IANN_A_1988696_SM0918.docx]

**Supplementary Information**

**Hereditary Transthyretin-Related Amyloidosis is frequent in polyneuropathy and cardiomyopathy of no obvious etiology**

A list of all Ethics committees that have approved the study for all participating centres in Germany, Austria and Switzerland is provided below.

**Germany:**

Ethik-Kommission bei der Landesärztekammer Baden-Württemberg

Ethikkommission der Medizinischen Fakultät, Universität Heidelberg

Ethik-Kommission an der Medizinischen Fakultät der Eberhard-Karls-Universität und am Universitätsklinikum Tübingen

Ethikkommission der Universität Ulm

Ethikkommission der Med. Fakultät der LMU München

Ethikkommission der Fakultät für Medizin der Technischen Universität München

Ethik-Kommission der FAU, Medizinischen Fakultät der Universität Erlangen-Nürnberg

Ethikkommission an der Universität Regensburg

Landesärztekammer Brandenburg Ethikkommission

Ärztekammer Bremen Ethikkommission

Ethik-Kommission der Ärztekammer Hamburg

Ethik-Kommission bei der Landesärztekammer Hessen

Ethik-Kommission des Fachbereichs Medizin der Justus-Liebig-Universität Gießen

Ethikkommission an der Medizinischen Fakultät der Universität Rostock

Ethikkommission der Medizinischen Hochschule Hannover

Ärztekammer Niedersachsen Ethikkommission

Ethikkommission der Universitätsmedizin Göttingen

Ethik-Kommission an der Med. Fakultät der RWTH Aachen am Universitätsklinikum Aachen

Ethikkommission der Medizinischen Fakultät der Heinrich-Heine-Universität Düsseldorf

Ärztekammer Nordrhein Ethikkommission

Ethikkommission der Ärztekammer Westfalen-Lippe und der Westfälischen Wilhelms-Universität Münster

Landesärztekammer Rheinland-Pfalz Ethik-Kommission

Sächsische Landesärztekammer Ethikkommission

Universität Leipzig Ethik-Kommission an der Medizinischen Fakultät

Ethik-Kommission der Medizinischen Fakultät der Martin-Luther-Universität Halle-Wittenberg

Ärztekammer Schleswig-Holstein, Ethik-Kommission

Universität zu Lübeck Ethik-Kommission

Universität Jena Ethik-Kommission

Landesärztekammer Thüringen Ethik-Kommission

**Austria:**

Ethikkommission der Landes Oberösterreich Keppler Universitätsklinikum

**Switzerland:**

Ethikkommission Nordwest- und Zentralschweiz

**Table 1S**. TRAM Study Group Participating Sites

| No. | Participating Sites | Principal Investigator |
| --- | --- | --- |
| 1 | Aachen, Universitätsklinikum Aachen, Klinik für Neurologie | Dr. Andrea Maier |
| 2 | Altenberg, Klinikum Altenburger Land GmbH, Klinik für Neurologie | Prof. Dr. Jörg Berrouschot |
| 3 | Arnsdorf, Sächsisches Krankenhaus Arnsdorf, Klinik für Neurologie und Neurologische Intensivmedizin | Prof. Dr. Tobias Back |
| 4 | Aschaffenburg, Gemeinschaftspraxis Neurologie und Psychiatrie im Stadtpalais Aschaffenburg | Dr. Jochen C. Ulzheimer |
| 5 | Bad Nauheim, Kerckhoff-Klinik GmbH, Klinik für Kardiologie | Dr. Andreas Rieth |
| 6 | Bad Neustadt, Rhön-Klinikum, Klinik für Neurologie und Neurologische Intensivmedizin | Dr. Hassan Soda |
| 7 | Berlin, Universitätsmedizin Charité, Klinik für Neurologie | Prof. Dr. Katrin Hahn |
| 8 | Berlin, Universitätsmedizin Charité, Medizinische Klinik mit Schwerpunkt Kardiologie und Angiologie, Prof. Dr. Fabian Knebel | Prof. Dr. Carsten Tschöpe |
| 9 | Bielefeld, Neurologische Klinik Bethel, Evangelisches Klinikum Bethel gGmbH | Prof. Dr. Wolf-Rüdiger Schäbitz |
| 10 | Bielefeld, Klinikum Bielefeld, Klinik für Kardiologie und internistische Intensivmedizin | Prof. Dr. Christoph Stellbrink |
| 11 | Bremen, Gesundheit Nord gGmbH, Klinik für Neurologie, Klinikum Bremen-Mitte | Prof. Dr. Andreas Kastrup |
| 12 | Buchholz, Krankenhaus Buchholz, **Sektion Kardiologie und Angiologie** | **Dr. Klaus Hertting** |
| 13 | **Buchholz,** Krankenhaus Buchholz, Fachabteilung Neurologie | Dr. Felix Butscheid |
| 14 | Celle, Allgemeines Krankenhaus Celle, Neurologische Klinik | Prof. Dr. Wolfgang Heide |
| 15 | Datteln, St. Vincenz Krankenhaus Datteln, Medizinische Klinik II | Dr. Marcus Bauer |
| 16 | Dortmund, Stroke-Unit und Neurologische Intensivstation, Klinikum Dortmund gGmbH | Dr. Gernot Reimann |
| 17 | Düsseldorf, LVR Klinikum Düsseldorf, Neurologie | Prof. Dr. Rüdiger Seitz |
| 18 | Eberswalde, Martin-Gropius-Krankenhaus, Klinik für Neurologie | Dr. Albert Grüger |
| 19 | Erlangen, Universitätsklinikum Erlangen, Medizinische Klinik2, Kardiologie und Angiologie | Prof. Dr. Stephan Achenbach |
| 20 | Essen, Alfried-Krupp-Krankenhaus, Klinik für Neurologie | Prof. Dr. Markus Krämer |
| 21 | Essen, Klinik für Kardiologie und Angiologie, Contilia Herz- und Gefäßzentrum, Elisabeth-Krankenhaus Essen | Dr. Robert Schueler |
| 22 | Friedberg, Kliniken an der Paar Friedberg Krankenhaus – Kardiologie | Dr. Heiko Methe |
| 23 | Fulda, Herz-Thorax-Zentrum Fulda, Klinikum Fulda AG, Universitätsmedizin Marburg - Campus Fulda | Prof. Dr. Volker Schächinger |
| 24 | Gießen, Neurologische Klinik des Universitätsklinikums Gießen und Marburg GmbH, Standort Gießen | Dr. Martin Jünemann |
| 25 | Göttingen, Universitätsmedizin Göttingen – Herzzentrum | Dr. Frauke Czepluch |
| 26 | Greifswald, Klinik und Poliklinik für Neurologie, Universitätsklinikum Greifswald der Ernst-Moritz-Arndt-Universität | Prof. Dr. Ulf Schminke |
| 27 | Haar bei München, kbo-Isar-Amper-Klinikum München-Ost, Klinik für Neurologie | Prof. Dr. Martin Marziniak |
| 28 | Halle, Universitätsklinik und Poliklinik für Innere Medizin III | Prof. Dr. Michel Noutsias |
| 29 | Halle, Universitätsklinik und Poliklinik für Neurologie, Universitätsklinikum Halle | Dr. Bernhard Sehm |
| 30 | Hamburg, Albertinen-Krankenhaus, Kardiologie | Prof. Dr. Herbert Naegele |
| 31 | Hamburg, Asklepios Klinik Altona-Neurologische Abteilung | Dr. Robert Berger |
| 32 | Hamburg, Neurologie Neuer Wall, Fachärzte für Neurologie & Psychiatrie | Dr. Karl Christian Knop |
| 33 | Hamburg, Universitäres Herzzentrum Hamburg, Klinik für Allgemeine und Interventionelle Kardiologie, Universitätsklinikum Hamburg-Eppendorf | Prof. Monica Patten-Hamel |
| 34 | Hamburg, Asklepios Klinik St. Georg, Zentrum für Neurologie | Dr. Christoph Terborg |
| 35 | Hannover, Medizinische Hochschule Hannover, Klinik für Neurologie | Prof. Dr. Thomas Skripuletz |
| 36 | Heidelberg, Heidelberg Universitätsklinikum, Klinik für Kardiologie, Angiologie und Pneumologie | Dr. Fabian Linden |
| 37 | Hoyerswerda, Lausitzer Seenland Klinikum GmbH, Klinik für Neurologie | Dr. Andreas Linsa |
| 38 | Jena, Universitätsklinikum Jena, Klinikum für Neurologie | Prof. Dr. Otto W. Witte |
| 39 | Jena, Universitätsklinikum Jena – Kardiologie | Prof. Dr. Paul Christian Schulze |
| 40 | Kassel, DRK-Kliniken Nordhessen GmbH, Klinik für Neurologie und Klinische Neurophysiologie | Dr. Christian Roth |
| 41 | Krefeld, Klinik für Neurologie, Krankenhaus Maria-Hilf \| Akademisches Lehrkrankenhaus der Heinrich-Heine-Universität Düsseldorf | Prof. Dr. Hans-Jürgen von Giesen |
| 42 | Leipzig, Klinische Neurophysiologie, Universitätsklinikum Leipzig - AöR, Klinik und Poliklinik für Neurologie | Prof. Dr. Petra Baum |
| 43 | Linz, Kepler Universitätsklinikum, Klinik für Interne 1 - Kardiologie und internistische Intensivmedizin | Dr. Clemenz Steinwender |
| 44 | Linz, Kepler Universitätsklinikum, Neurologie, Neuromed Campus | Dr. Tim von Oertzen |
| 45 | Lübben, Asklepios Fachklinikum Lübben, Abteilung für Neurologie | Prof. Dr. Andreas Bitsch |
| 46 | Luzern, Luzerner Kantonspital, Kardiologie | Dr. Richard Kobza |
| 47 | Lübeck, Medizinische Klinik II (Kardiologie, Angiologie, Intensivmedizin), Universitäres Herzzentrum Lübeck | Dr. Tobias Graf |
| 48 | Lübeck, Sana Kliniken Lübeck GmbH, Medizinische Klinik II | Prof. Dr. Joachim Weil |
| 49 | Mainz, DRK Schmerz-Zentrum | Dr. Christian Geber |
| 50 | Mainz, Johannes-Gutenberg-Universität Mainz, Klinik und Poliklinik für Neurologie | Prof. Dr. Frank Birklein |
| 51 | Mannheim, Diakonissen Speyer-Mannheim, Diakonissenkrankenhaus Mannheim, Klinik für Neurologie | Dr. Joachim Wolf |
| 52 | Mosbach, Neckar-Odenwald Kliniken gGmbH, Kreiskrankenhaus Mosbach, Kardiologie der Klinik für Innere Medizin | Dr. Peter A. Oberst |
| 53 | Mühlhausen, Hainich Klinikum Mühlhausen, Neurologische Klinik und Poliklinik | Prof. Dr. Marek Jauss |
| 54 | München, Klinik und Poliklinik für Neurologie, Klinikum rechts der Isar der TU München | Prof. Dr. Achim Berthele |
| 55 | München, Kardiologie München Nord | Dr. Hans Ullrich Ebersberger |
| 56 | München, Friedrich-Baur-Institut, Neurologische Klinik und Poliklinik, Ludwig-Maximilians-Universität | Dr. Beate Schlotter-Weigel |
| 57 | München, Neurologische Klinik und Poliklinik & Deutsches Schwindel- und Gleichgewichtszentrum DSGZ, Ludwig-Maximilians-Universität München | Prof. Dr. Andreas Straube |
| 58 | München, Praxis für Neurologie | Dr. Holger Albrecht |
| 59 | Neuruppin, Ruppiner Kliniken GmbH, Hochschulklinikum der MHB, Klinik für Neurologie | Dr. Tobias Müller |
| 60 | Olten, Kantonsspital Olten - Solothurner Spitäler, Kardiologie | Prof. Dr. Rolf Vogel |
| 61 | Perleberg, Kreiskrankenhaus Prignitz GmbH | Dr. Marko Petrick |
| 62 | Regensburg, Krankenhaus Barmherzige Brüder Regensburg , Klinik für Neurologie | Dr. Lukas Kremmler |
| 63 | Regensburg, Universitätsklinikum Regensburg, Kardiologie | Prof. Dr. Bernhard Unsöld |
| 64 | Sande, Neurologische Klinik, Nordwest-Krankenhaus Sanderbusch gGmbH | Prof. Dr. Pawel Kermer |
| 65 | Schkeuditz, Sächsisches Krankenhaus Altscherbitz, Fachkrankenhaus für Psychiatrie und Neurologie | Dr. Anne-Dorte Sperfeld |
| 66 | Siegen, Jung-Stilling Klinikum Siegen, Diakonie Westfalen Süd, Neurogeriatrie | Prof. Dr. Christian Tanislav |
| 67 | Stadtlohn, Krankenhaus und MVZ Maria-Hilf Stadtlohn GmbH, Kardiologie | Dr. Alessandro Cuneo |
| 68 | Teupitz, Asklepios Fachklinikum Teupitz, Abteilung für Neurologie | Prof. Dr. Andreas Bitsch |
| 69 | Trier, Krankenhaus der Barmherzige Brüder Trier, Neurologie und Neurophysiologie | Prof. Dr. Matthias Maschke |
| 70 | Tübingen, Universitätsklinikum Tübingen, Zentrum für Neurologie, Neurologische Klinik, Poliklinik und Hertie-Institut für klinische Hirnforschung | Prof. Alexander Grimm |
| 71 | Ulm, NeuroPoint GmbH | Prof. Dr. Herbert Schreiber |
| 72 | Ulm, Universitätsklinikum Ulm, Klinik für Neurologie | Prof. Dr. Albert C. Ludolph |
| 73 | Weimar, Sophien-u.Hufeland-Klinikum, Klinik für Neurologie und Klinische Neurophysiologie | Dr. Rolf Malessa |
| 74 | Wermsdorf, Fachkrankenhaus Hubertusburg gGmbH, Neurologische Klinik | Dr. Adrian Trommer |
| 75 | Wismar, Sana HANSE Klinikum Wismar, Klinik für Neurologie | Dr. Sabine Mehnert |
| 76 | Wolfratshausen, Praxis für Neurologie | Dr. Michaela Krause |
| 77 | Zug, Praxisgemeinschaft für Kardiologie, Innere Medizin und Psychosomatische Medizin | Dr. Georges Borek |
